# An FD-LC-MS/MS Proteomic Strategy for Revealing Cellular Protein Networks: A Conditional Superoxide Dismutase 1 Knockout Cells

**DOI:** 10.1371/journal.pone.0045483

**Published:** 2012-09-18

**Authors:** Tomoko Ichibangase, Yasuhiro Sugawara, Akio Yamabe, Akiyo Koshiyama, Akari Yoshimura, Takemi Enomoto, Kazuhiro Imai

**Affiliations:** Research Institute of Pharmaceutical Sciences, Musashino University, Tokyo, Japan; Shantou University Medical College, China

## Abstract

Systems biology aims to understand biological phenomena in terms of complex biological and molecular interactions, and thus proteomics plays an important role in elucidating protein networks. However, many proteomic methods have suffered from their high variability, resulting in only showing altered protein names. Here, we propose a strategy for elucidating cellular protein networks based on an FD-LC-MS/MS proteomic method. The strategy permits reproducible relative quantitation of differences in protein levels between different cell populations and allows for integration of the data with those obtained through other methods. We demonstrate the validity of the approach through a comparison of differential protein expression in normal and conditional superoxide dismutase 1 gene knockout cells and believe that beginning with an FD-LC-MS/MS proteomic approach will enable researchers to elucidate protein networks more easily and comprehensively.

## Introduction

Systems biology is an emerging field that aims to understand biological phenomena in terms of complex biological and molecular interactions. So-called “-omics” technologies, such as proteomics, are the major forces that drive systems biology by providing researchers with information regarding protein networks [1], [2]. However, in recent years, investigators have become wary of -omics approaches because the data they generate may be limited by false negative/positive results. Since the decisions most likely arise as a result of variance in the data, there is great need to develop more precise, reproducible, and quantitative analytical methodologies for use in systems biology [3], [4].

**Figure 1:**
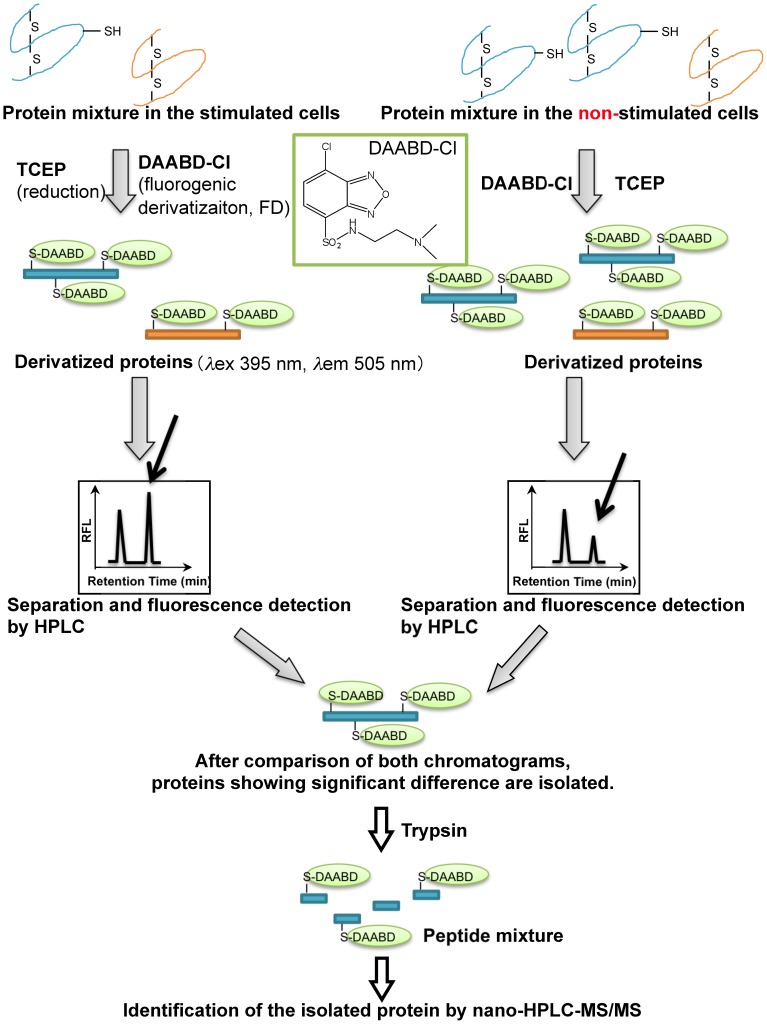
Schematic illustration of the FD-LC-MS/MS proteomic method. After fluorogenic derivatization, the protein mixtures are separated by HPLC, and proteins exhibiting significant differential expression are isolated and identified using nano-HPLC-MS/MS and database searching.

Over the past few years, we have developed a reproducible and quantitative proteomic methodology for identifying biomarkers by elucidating differences in protein expression between differing cell types, such as breast cancer cells and normal cells (Fig. 1) [5]. The method, known as fluorogenic derivatization-liquid chromatography-tandem mass spectrometry FD-LC-MS/MS, involves derivatizing proteins with the fluorogenic derivatization reagent DAABD-Cl (7-chloro-N-[2-(dimethylamino)ethyl]-2,1,3-benzoxadiazole-4-sulfonamide), followed by separation of the derivatized proteins using high-performance liquid chromatography (HPLC) with fluorescence detection. Extracts prepared from cells cultured under different conditions are subjected to FD-LC-MS/MS and the resulting chromatograms are compared in order to identify peaks of significantly differing heights that represent differentially expressed proteins. The differentially expressed proteins are then isolated and subjected to enzymatic digestion and identification using nano-HPLC combined with tandem mass spectrometry and a database-searching algorithm. The features of DAABD-Cl are its hydrophilicity and thiol-specific fluorogenic property, and the derivatized proteins also keep their hydrophilicity and thus do not precipitate after complete derivatization of proteins. Moreover, relatively small size of the reagent molecules enable it to react completely with cysteine residues in the proteins under a mild condition, and their derivatives are highly fluorescent with longer emission wavelength than natural fluorescence of biomolecules (less than 400 nm), affording the sensitive detection of the protein derivatives. The method is highly sensitive (femtomole-level detection) due to less noisy fluorogenic rather than fluorescence derivatization, and enables precise and comprehensive relative quantitation of protein levels (ca. 3.8 and 20% between-day relative standard deviation of peak heights for lactalbumin and biological samples, respectively) by combining FD with HPLC separation [6]. The FD-LC-MS/MSmethod requires only a minimal amount of sample. The FD-LC-MS/MS method has the advantage over gel- and other MS-based proteomic methods for assessing relative protein expression levels in that it does not require calibrating raw data of replicated experiments. Gel- and MS-based analyses must calibrate the raw data due to their high variability, and these added steps tend to increase the chances of false results. Furthermore, in our previous study using breast cancer cells, in addition to the finding of the biomarkers, obtained data contributed to the understanding of the events leading to the progression of breast cancer [5]. Based on the experience, we learn to know the fact that the reproducible, quantitative and comprehensive method, FD-LC-MS/MS, could be a tool to predict protein networks.

**Figure 2:**
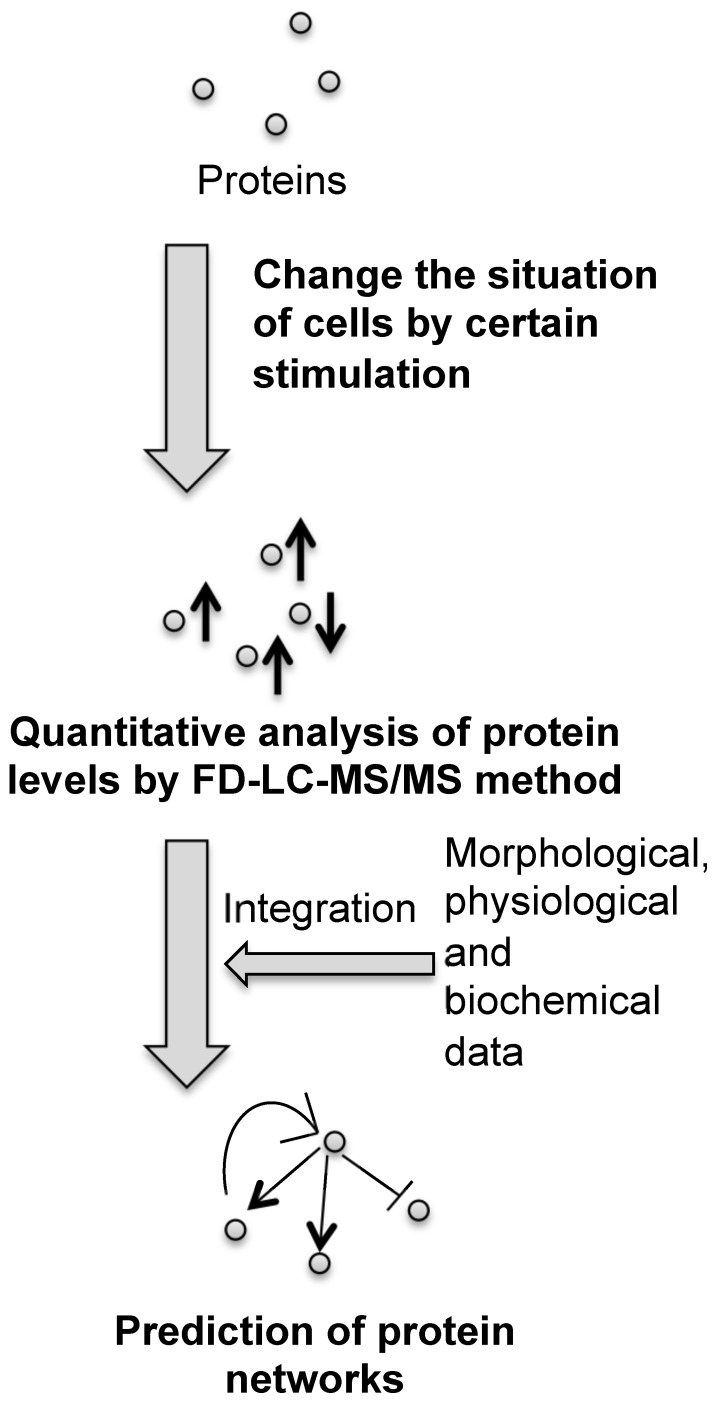
Simplified scheme for the proposed strategy. Using the method, protein expression changes caused by a particular stimulus are quantified. The resulting data are integrated with other data and a diagram of predicted protein networks is constructed.

Here, we propose a novel strategy for identifying protein networks in cells using the FD-LC-MS/MS method (Fig. 2). Cells are initially cultured under conditions that will allow protein networks usually hidden by homeostasis to emerge. Proteins that are differentially expressed are then relatively quantified and identified using FD-LC-MS/MS. The resulting data are integrated with data obtained using other distinct approaches such as morphological observations, and protein networks are then predicted. To evaluate this strategy, we used conditional superoxide dismutase 1 (SOD1)-knockout chicken DT40 (SOD1(–)) cells in which the expression of the SOD1 gene can be turned off by treating the cells with doxycycline (Dox). Eventually, the data obtained using the FD-LC-MS/MS proteomic strategy described here may enable us to obtain a more comprehensive understanding of the changes in cellular protein networks that occur in response to various stressors, such as the depletion of SOD1.

## Results

### Optimization of HPLC-fluorescence conditions

The conditions for HPLC separation were optimized for use in these experiments based on conditions used in a previous study of liver samples [7]. The optimized mobile phases and the final gradient elution program were shown in the Materials and Methods section. On the typical chromatograms (Fig. S1), the reproducibility of the HPLC method was calculated using the height of peaks 15, 35, and 29 as representatives of high, medium, and low peaks, respectively. The between-day relative standard deviation (RSD, %) was less than 0.6% (high peak), 3.3% (medium peak), and 21.8% (low peak) (n>3).

### Differential proteomic analysis

Proteins extracted from SOD1(−) cells treated with Dox for 96 h were derivatized with DAABD-Cl and separated using HPLC with fluorescence detection. As a control, proteins extracted from SOD1(+) cells were separately derivatized and subjected to HPLC with fluorescence detection. The resulting chromatograms are shown in Figure S1. The height of each peak detected in the chromatogram of SOD1(−) cell proteins was compared with that of the corresponding peak in the chromatogram of proteins from SOD1(+) cells (Table S1). Fractions associated with only those peaks for which the height differed significantly between the SOD1(−) and SOD1(+) samples were isolated, digested with trypsin, and identified using nano-HPLC-MS/MS. The resulting MS data are shown in Table S2.

**Figure 3:**
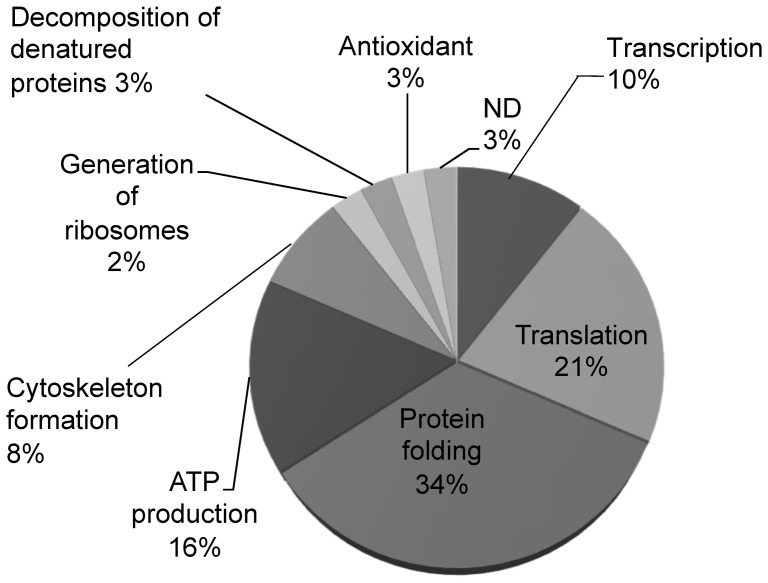
Classification of identified proteins. Functional classification of differentially expressed proteins identified in this study. The most significant changes were observed with proteins involved in mediating protein folding. ND: No Data.

To quantify changes in the levels of cellular proteins, the height of each peak (indicative of the level of protein) in chromatograms obtained from SOD1(−) cell lysates was compared to the height of the corresponding peak in chromatograms obtained from SOD1(+) cell lysates. Proteins that were identified from fractions corresponding to peaks that differed significantly (*p*<0.05) in height are listed and classified by function in Table S1 and Fig. 3. The height of 37 peaks (from which 23 proteins were identified as described in the Materials and Methods section and functionally classified) in chromatograms of SOD1(−) cell lysates differed significantly from the height of corresponding peaks in chromatograms of SOD1(+) cell lysates. An analysis using the protein nucleotide-nucleotide-basic local alignment search tool (Protein Blast) indicated that the gene encoding hypothetical protein RCJMB04_5f14 (gi|57525441) (peak 11 on Table S1) shared complete sequence homology with the gene encoding destrin (100%, gi|118463); therefore, this protein was classified as being involved in cytoskeleton formation. There are no reports describing the function of either 40s ribosomal protein S4 (40s RP S4) or ubiquitin-40s ribosomal protein S27u (Ub-40s RP S27u).

## Discussion

More than 280 well-resolved protein peaks were detected under optimized conditions, and the reproducibility of the chromatographic peak heights was similar to that obtained in a previous paper in which breast cancer cells were examined [5].

Since the method does not disrupt peptide modifications, the detection of post-translational modifications and isomers is possible using this method. Therefore, it is possible that peaks with different retention times identified as the same protein could represent post-translational modifications. However, at present it is difficult to identify post-translational modifications using nano-HPLC-MS combined with collision-induced dissociation (CID) of the peptides.

**Figure 4:**
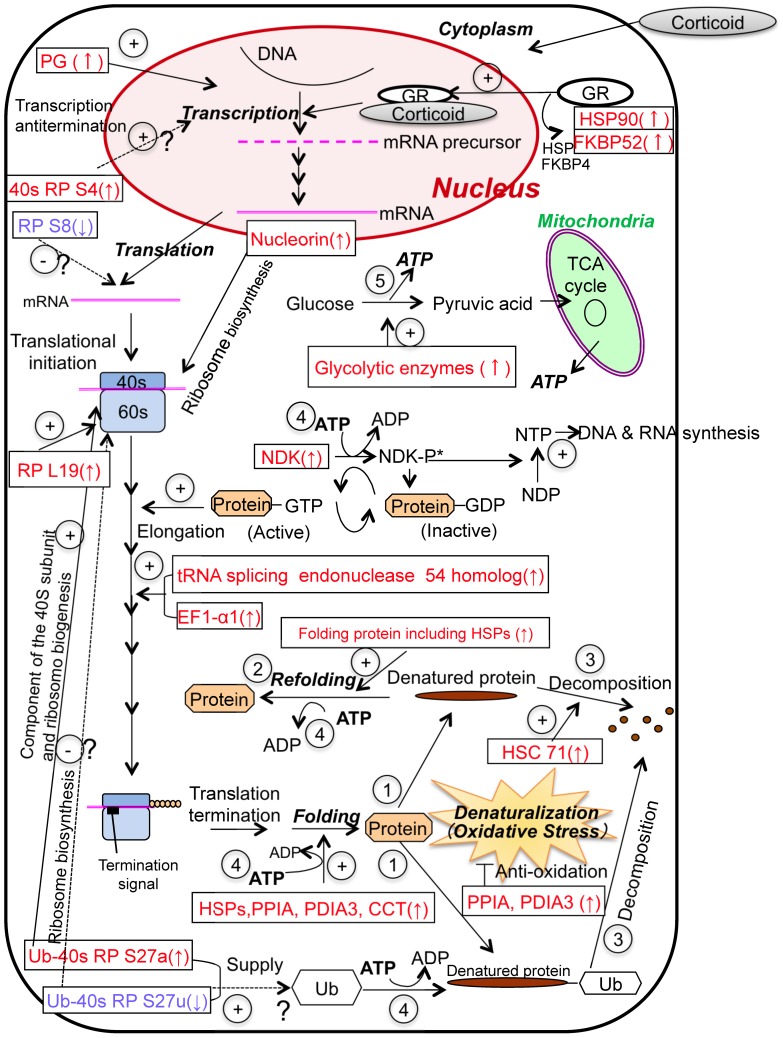
Predicted protein networks in SOD1(−) cells. Protein networks for denaturation, refolding, decomposition, ATP-consumption and -production. Arrows indicate increased (up) and decreased (down) expression. The (+) and (−) signs indicate enhancement or suppression, respectively, of cellular processes in response to changes in protein expression. The abbreviated protein names are in Table S1. GR: glucocorticoid receptor.

**Figure 5:**
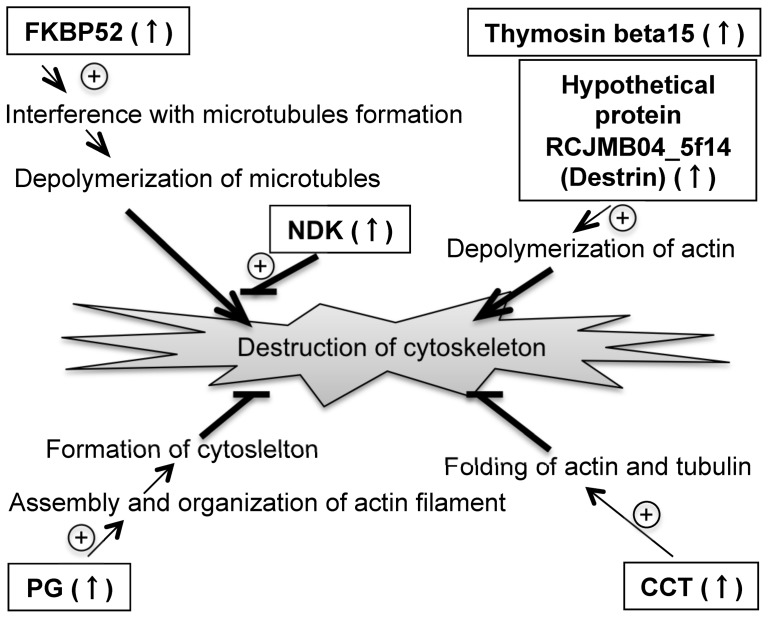
Flow diagram of protein networks involved in the assembly and disassembly of cytoskeleton. Increased expression of PG, NDK, and CCT mitigates the effects of cytoskeletal disassembly, while increased expression of FKBP52, destrin, and thymosin beta 15 promotes disassembly.

Classification of the identified proteins according to function (Fig. 3) indicated that the expression of proteins involved in transcription, translation, protein folding, ATP production and cytoskeleton formation was altered by depletion of SOD1. Considering the FD-LC-MS/MS results in conjunction with morphological, physiological and biochemical data, we propose several possible protein networks (Figs. 4 and 5). Since the expression of proteins involved in protein folding (e.g., the heat shock family of proteins) and the decomposition of denatured proteins (e.g., heat shock cognate 71 kDa protein (HSC71)) significantly increased in SOD1(−) cells (Table S1), it is likely that increased oxidative stress resulted in protein damage (arrow in Fig. 4), initiating a response that involved proper refolding (arrow) or decomposition (arrow) of the damaged proteins. The response suggests that the cells adapted to the increased oxidative stress resulting from depletion of SOD1.

The expression of proteins involved in transcription and translation was also significantly upregulated in SOD1(−) cells, probably to counteract the effects of protein decomposition in order to maintain cellular homeostasis. During protein synthesis, GTP is required as an energy source. The enzyme nucleoside diphosphate kinase (NDK) mediates the generation of GTP from GDP, and NDK expression was upregulated in SOD1(−) cells, using ATP (arrow, Fig. 4). Additionally, the expression of several glycolytic proteins, including fructose-bisphosphate aldolase C, pyruvate kinase muscle isozyme, phosphoglycerate mutase 1, and phosphoglycerate kinase was upregulated in SOD1(−) cells (arrow, Fig. 4). Increased expression of these enzymes should activate the TCA cycle in order to meet the increased demand for ATP.

Microscopic observation of SOD1(−) cells revealed the presence of cytoskeletal abnormalities, including damaged microtubules (Fig. S2). In agreement with this observation, FD-LC-MS/MS analysis of SOD1(−) cell extracts indicated an increase in the expression of proteins involved in cytoskeleton formation, including thymosin beta 15, hypothetical protein RCJMB04_5f14 (destrin), peptidyl-prolyl cis-trans isomerase FKBP4 (FKBP52), plakoglobin (PG; classified as a transcription-related protein in Table S1), T-complex protein 1 subunit zeta (CCT; classified as being involved in protein folding in Table S1), and NDK (classified as being involved in ribosome generation in Table S1). As shown in Fig. 5, thymosin beta 15 and destrin are involved in depolymerization of actin filaments, and FKBP52 is known to interfere with microtubule formation. On the other hand, PG, CCT, and NDK are involved in maintenance of the actin cytoskeleton, actin and tubulin folding, and microtubule polymerization via the production of GTP, respectively.

The rest of descriptions on cellular responses to oxidative stress based on the alteration of protein levels in SOD1(−) cells were given in Text S1.

In summary, we developed a novel strategy for integrating data obtained from FD-LC-MS/MS and morphological, physiological and biochemical approach to elucidate hidden protein networks in cells. Protein expression in normal and SOD1-deficient cells was compared to demonstrate how our FD-LC-MS/MS proteomic strategy could be used to examine the effects of particular mutations (in this case, one that leads to increased oxidative stress) or other cellular changes (e.g., transformation) on protein networks. The FD-LC-MS/MS method provides comprehensive and quantitative data regarding relative protein expression, and can be used to examine changes in the expression of a single protein or an entire protein network. Although numerous approaches are typically employed in the study of complex biological phenomena from a systems biology perspective, we believe that beginning with an FD-LC-MS/MS proteomic approach will enable researchers to elucidate protein networks more easily and comprehensively.

## Materials and Methods

### Generation and culture of *SOD1* knockout cells

The generation of the *SOD1* knockout cells is described elsewhere [8]. Cells were cultured at 39°C in RPMI-1640 medium supplemented with 10% fetal bovine serum, 1% chicken serum, and 100 μg/mL kanamycin in the presence or absence of 1.0 μg/mL Dox. The level of the SOD1 protein in these cells reaches the lower limit of detection 96 h after Dox addition [8].

### Sample preparation and calculation of cell numbers

Cell numbers were determined using a hemocytometer. Cells (4–6×10^7^) were collected by centrifugation and washed with PBS three times. The final cell pellet was resuspended with three volumes of 10 mM CHAPS and maintained on ice until the cells were homogenized with 10 strokes in a Dounce homogenizer. The lysate was centrifuged at 20,400× *g* for 15 min at 4°C, after which the supernatant was recovered for use in experiments.

### FD and HPLC conditions

All experiments were repeated at least three times. Detailed conditions for the FD reaction and protein quantitation using HPLC are described elsewhere [5]. Briefly, 10 μL of cell lysate (4.6×10^5^ cells diluted with 10 mM CHAPS) was mixed with 60 μL of a solution containing 0.83 mM tris (2-carboxyethyl) phosphine hydrochloride (TCEP; Sigma), 3.3 mM ethylenediamine-N,N,N',N'-tetraacetic acid sodium salt (Na_2_EDTA; Wako), 17 mM CHAPS (Dojindo) in 6.0 M guanidine hydrochloride buffer (pH 8.7) (Tokyo Chemical Industry), 25 μL of the buffer, and 5.0 μL of 140 mM DAABD-Cl (TCI) in acetonitrile. The reaction mixture was placed in a 40°C water bath for 10 min and then 3.0 μL of 20% trifluoroacetic acid (TFA; Wako) was added to stop the derivatization reaction. A total of 20 μL of the reaction mixture was injected into a Hitachi L-2000 series HPLC system (λex, 395 nm; λem, 505 nm) at a flow rate of 0.55 mL/min. Proteins were separated on an Intrada WP-RP column (250×4.6 mm i.d., Imtakt Co.) maintained at 60°C. The mobile phases consisted of 0.20% TFA in acetonitrile/isopropanol/water (9.0/1.0/90; A) and 0.15% TFA in acetonitrile/isopropanol/water (69/1.0/30; B). The gradient program was as follows: 5.0% B held for 15 min; to 30% B by 30 min and then held at 30% B for 65 min; to 35% B by 75 min; to 38% B by 135 min; to 40% B by 175 min; to 40.5% B by 195 min; to 44% B by 215 min and held until 245 min; to 60% B by 505 min; to 100% B by 585 min and held until 600 min.

All reagents were of analytical grade and were used without further purification. Water was used after purification with the Milli-Q system (Nihon Millipore). Chromatography data acquisition, processing and control of the LC system utilized EZ chrom elite software, version 3.1.7J. Peaks were correlated between chromatograms based upon the specific retention time of the derivatives and were confirmed by isolation and identification of the derivatives.

### Identification of derivatized proteins using database searching

The isolated derivatized proteins were identified using nano-HPLC and tandem mass spectrometry as described in a previous report [5]. Each peak fraction obtained from the HPLC separation was concentrated to 5.0 μL under reduced pressure. The residue was diluted with 50 μL of 50 mM ammonium bicarbonate (pH 7.8; Sigma) containing 10 mM calcium chloride (Wako) and 0.50 U of trypsin (Promega) and incubated for 2.0 h at 37°C. The resulting peptide mixture (20 μL) was directly subjected to nano-HPLC separation on an Ultimate 3000 system (Dionex) connected to a hybrid LTQ-Orbitrap XL mass spectrometer (Thermo Fisher Scientific). The sample was loaded from the injection loop to a nano-precolumn (300 μm i.d. ×5.0 mm, C18 PepMap, LC Packings) and washed with 0.10% TFA in 2.0% acetonitrile for 5.0 min at a flow rate of 25 μL/min. Peptides were then separated on a nano-HPLC capillary column (75 μm i.d. ×3.0 μm, Nikkyo Technos) at a flow rate of 0.30 μL/min employing a gradient from 0–40% buffer B (0.10% formic acid in 80% acetonitrile) over a period of 35 min. Buffer A consisted of 0.10% formic acid in 2.0% acetonitrile. Peptides were eluted into the MS with a capillary voltage 45 V and a spray voltage ranging from 1.2–1.9 kV. The transfer capillary temperature was set at 200°C. Data-dependent acquisition was controlled using Instrument Setup software, version 2.0.7. SP1 (Thermo Fisher Scientific). The acquisition cycle consisted of a survey scan covering the m/z range 350–1500, followed by MS/MS fragmentation of the five most intense precursor ions. Spectra were acquired under automated control with a maximal ion injection time of 500 ms. For accurate mass measurements, the Lock Masses were set at m/z  = 391 and 445. The amino acid sequence data resulting from automated interpretation of MS/MS spectra were searched against the National Center for Biotechnology Information non-redundant (NCBInr 2011.11.07_15,916,306 sequences; 5,467,648,827 residues_Taxonomy: bony vertebrates (1,443,524 sequences)) database using MASCOT version 2.3.01 (Matrix Science). Trypsin and bony vertebrates were selected as the enzyme and taxonomy, respectively, and one missed cleavage was allowed. Variable modification of cysteine residues with DAABD was selected, but no fixed modifications were allowed. The mass tolerance and the fragment ion mass tolerance were set at 0.50 ppm and 0.70 Da, respectively. If multiple proteins matched a set of peptides, the highest scoring protein containing one or more cysteine residues (other than ubiquitous keratin) among the *Gallus gallus* as species was selected as the correct identification.

### Statistical analysis

Results are expressed as the mean ± SD. The significance of differences between means was determined using a two-tailed Student’s t test.

### Immunostaining

After suspending in cold-PBS, cells were applied to a 8-well Culture Slide (BD falcon) (2×105 cells/well), and then the slide was centrifuged at 500 rpm for 5 min at 4°C. Cells on the slide were dried and fixed with 3.7% formaldehyde for 15 min and than, permeabilized with 99.5% ethanol for 10 min. After washing with PBS, cells were incubated with 1 μg/mL Hoechst 33258 in PBS for 5 min and then, with 3% bovine serum albumin for 30 min. After washing three times with PBS, anti-α-tubulin antibody (1∶500) was added and incubated for 1.0 h. Subsequently, after washing three times with PBS, Cy3 conjugated anti-mouse IgG (Jackson immune research) was added and incubated for 1.0 h. After washing three times with PBS, the slide was mount with PermaFluor (Immunon), and cells were observed with a Zeiss Axiovision (Carl Zeiss). After dissolved in cold-PBS, the cells applied to 8-well Culture Slide (BD falcon) (2×10^5^ cells/well), and then the slides were centrifuged for 5 min at 500 rpm, 4°C. For immobilization, the slides dried and 3.7% formaldehyde was added for 15 min. The permeabilization step was performed with 99.5% ethanol for 10 min. After washing with PBS, Hoechst solution in PBS (1 μg/mL) were added and incubated for 5 min. And then, 3% bovine serum albumin was added in well for 30 min. After wash three times with PBS, anti-α-tubulin antibody (1∶500) was added and incubated for 1.0 h. Subsequently, wash three times wash with PBS, Cy3 conjugated α-mouse IgG (Jackson immune research) was added and incubated for 1.0 h. After wash three times with PBS and mount with PermaFluor (Immunon), the slides were inspected on a Zeiss Axiovision (Carl Zeiss). To clearly observe the cytoskeletal abnormalities, SOD1(−) cells treated with Dox for 132 h.

## Supporting Information

Figure S1 **Typical chromatograms derived from SOD1(−) and SOD1(+) cells.** Using fluorescence detection, more than 280 peaks representing derivatized proteins were observed from a single injection of an extract prepared from 9.2×10^4^ cells.
(PPTX)

Figure S2 **Immunostaining of α-tubulin.** To investigate the effect of SOD1 deficit for cytoskeleton, immunostaining of α-tubulin were performed in SOD1(−) and SOD1(+) cells. Cells were stained with Hoechst 33258 and immunostaied with an anti-α-tubulin andibody. Upper and lower pannels were images of SOD1(−) and SOD1(+) cells, respectively. The cytoskeletal abnormalities including damaged microtubules were observed in SOD1 (−) cells.
(PPT)

Table S1 **Proteins for which expression differed significantly (p<0.05) between SOD1(−) and SOD1(+) cells and the expression ratio.**
(XLSX)

Table S2 **Identified proteins differentially expressed in SOD1(−) and SOD1(+) cells.**
(XLSX)

Text S1 (DOC)
