# Supplementary figures and images for: An FD-LC-MS/MS Proteomic Strategy for Revealing Cellular Protein Networks: A Conditional Superoxide Dismutase 1 Knockout Cells
Source: PLoS One. 2012 Sep 18;7(9):e45483. doi: 10.1371/journal.pone.0045483 (PMC3445526; doi:10.1371/journal.pone.0045483)

## Slide 1
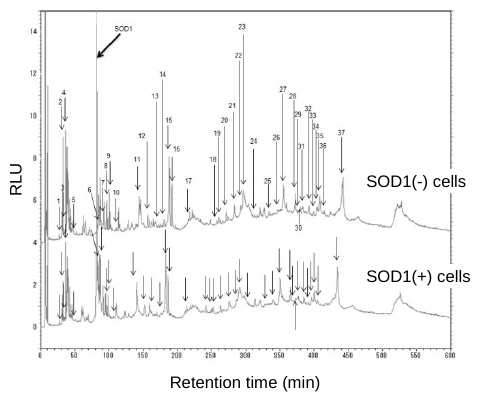

RLU
SOD1(-) cells
SOD1(+) cells
Retention time (min)

Supplement: Figure S1 — Typical chromatograms derived from SOD1(−) and SOD1(+) cells. Using fluorescence detection, more than 280 peaks representing derivatized proteins were observed from a single injection of an extract prepared from 9.2×10^4^ cells. (PPTX) [file pone.0045483.s001.pptx]

## Slide 1
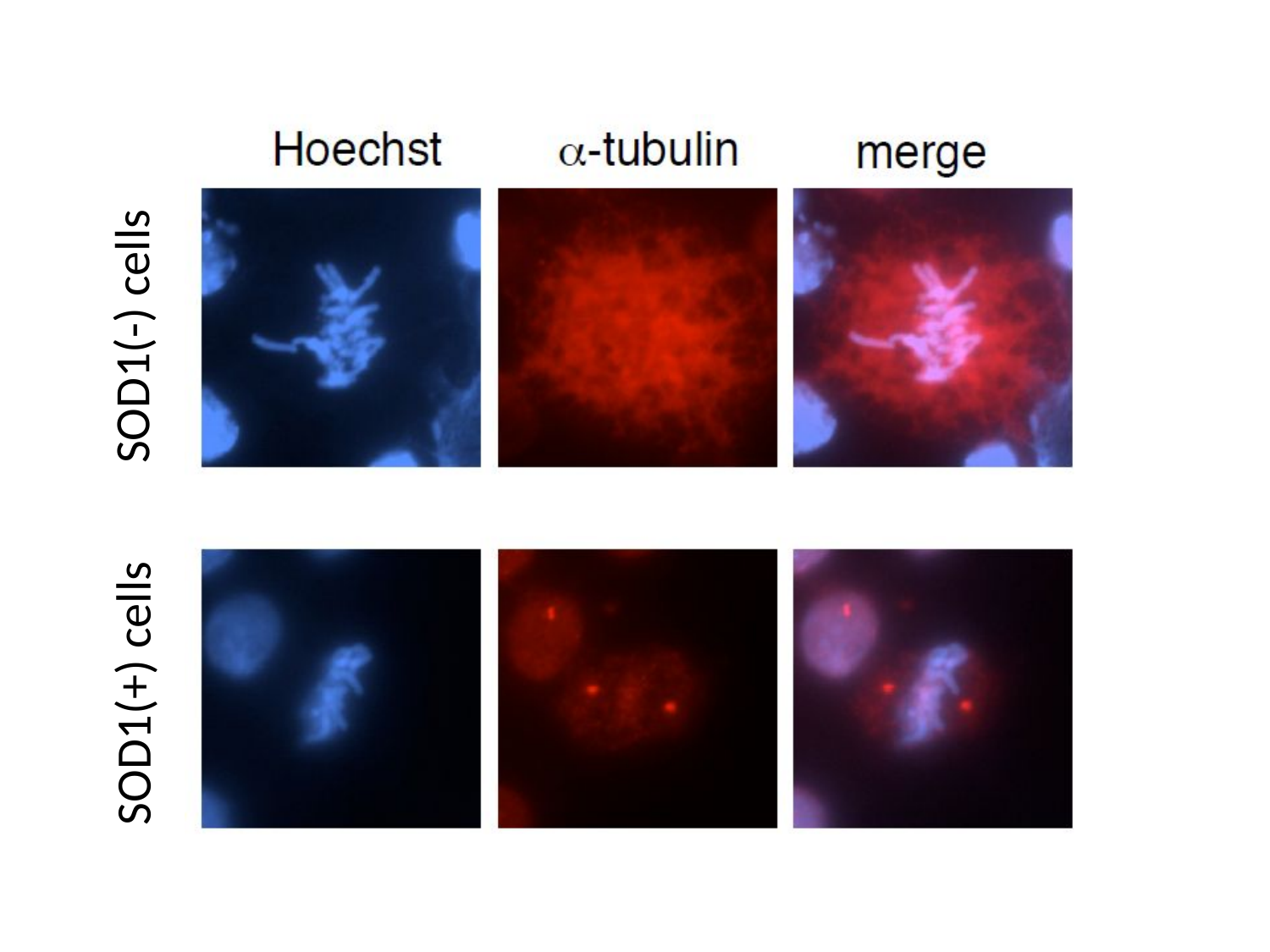

SOD1(-) cells
SOD1(+) cells

Supplement: Figure S2 — Immunostaining of α-tubulin. To investigate the effect of SOD1 deficit for cytoskeleton, immunostaining of α-tubulin were performed in SOD1(−) and SOD1(+) cells. Cells were stained with Hoechst 33258 and immunostaied with an anti-α-tubulin andibody. Upper and lower pannels were images of SOD1(−) and SOD1(+) cells, respectively. The cytoskeletal abnormalities including damaged microtubules were observed in SOD1 (−) cells. (PPT) [file pone.0045483.s002.ppt]
