# Supplementary material for: An FD-LC-MS/MS Proteomic Strategy for Revealing Cellular Protein Networks: A Conditional Superoxide Dismutase 1 Knockout Cells
Source: PLoS One. 2012 Sep 18;7(9):e45483. doi: 10.1371/journal.pone.0045483 (PMC3445526; doi:10.1371/journal.pone.0045483)
Supplement: Text S1 — (DOC) [file pone.0045483.s005.doc]

Considering alteration of protein levels in SOD1(-) cells upon depletion of SOD1, we can depict cellular responses to oxidative stress as follows. At first, plakoglobin (PG; γ-catenin), which is classified as being involved in transcription, is highly homologous to β-catenin, and is a component of cell-cell adherent junctions that link cadherin receptors to cytoskeletal actin, as well as β-catenin. It was reported that overexpression of PG results in elevated nuclear translocation of endogenous β-catenin, which activates transcription by forming a complex with the Lef/Tcf (lymphoid enhancing factor/T-cell factor) family transcription factor in Wnt signaling [1]. The observed increase in PG expression in SOD1(-) cells thus suggests that transcription mediated by Wnt signaling is upregulated in these cells.

In addition, both heat shock protein HSP 90-alpha (HSP90) and peptidyl-prolyl cis-trans isomerase FKBP4 (alternative name: 52 kDa FKBP (FKBP52)) cooperatively enhance transcription. FKBP52 forms an activated glucocorticoid receptor (GR) complex with HSP90 to transport GR from the cytoplasm to the nucleus, where it binds target DNA sequences as a transcription factor [2].

Secondly, we found that a number of proteins involved in translation are differentially regulated in SOD1(-) cells, including ubiquitin-40s ribosomal protein S27a (Ub-40s-RPS27a), tRNA splicing endonuclease 54 homolog, ribosomal protein L19 (RPL19), ribosomal protein S8 (RPS8), and elongation factor 1-alpha 1 (EF1-α1). Ub-40s-RPS27a and RPL19 are components of the 40s and 60s ribosomal subunits, respectively. Increased expression of these proteins may thus increase ribosome biogenesis, resulting in enhanced translation. Also, the Ub moiety of Ub-fusion proteins such as Ub-40s-RPS27a is known to be involved in facilitating ribosome biogenesis [3]. Though RPS8 is a component of the 40s ribosome, its precise function is still not clear. Either overexpression or knockdown of RPS8 reportedly results in the suppression of translation, suggesting that RPS8 is a rate-limiting factor in translational regulation [4]. Therefore, the remarkable decrease in the expression of RPS8 observed in SOD1(-) cells might affect the efficiency of translation. RPL19 reportedly functions as an RNA chaperone during translation, coordinating interactions between the ribosome and RNA [5]. Hence, increased expression of RPL19 might enhance translation. It has been also reported that expression of RPL19 is upregulated in several cancers [5], although what role this might play in cancer progression remains unclear. Both tRNA splicing endonuclease 54 homolog and EF1-α1 play important roles in polypeptide elongation [6], and increased expression of these proteins might enhance the efficiency of translation in SOD1(-) cells. In addition, increased expression of nucleolin and nucleoside diphosphate kinase (NDK) also seems to promote translation. Nucleolin is an abundant protein in the nucleolus, and is directly involved in the regulation of ribosome biogenesis and maturation. Numerous recent reports have shown that nucleolin functions as a transporter between the nucleus and the cytoplasm and plays an important role in regulating cell growth. NDK catalyzes the synthesis of nucleoside triphosphates (NTP) from nucleoside diphosphates (NDP) using ATP. NTP are used in the synthesis of DNA and RNA, and NDK-P*, which is the activated intermediate, provides the GTP required for protein synthesis and microtubule polymerization.

Thirdly, our results also indicate that a number of proteins involved in regulating protein folding are differentially expressed in SOD1(-) cells, including 10 kDa heat shock protein, mitochondrial (HSP10), peptidylprolyl isomerase A (PPIA; alterative name: cyclophilin A), protein disulfide-isomerase A3 precursor (PDIA3), HSP90, and T-complex protein 1 subunit zeta (alternative name: chaperonin-containing T-complex polypeptide 1 (CCT)). The HSPs, namely HSP10 and HSP90, are constitutively expressed in cells, but are overexpressed when cells are exposed to stressors, such as high levels of reactive oxygen species. Since HSPs act as intracellular chaperones, their increased expression in SOD1(-) cells would enable cells to maintain normal functions by repairing unfolded or misfolded proteins. The HSP10 protein was originally identified as a mitochondrial chaperone, and now is known to be present in the cytosol as well. It has been reported that HSP10 is abnormally released into the cytosol from mitochondria in malignant disorders, and the protein not only mediates folding of mitochondrial proteins in association with HSP60, but also is involved in the promotion of cell growth and suppression of apoptosis by protecting cells from stress [7]. In the present study, the heights of two of the three peaks identified as HSP10 were higher in SOD1(-) cells, though there was no fluctuation in the height of peaks derived from HSP60. This suggests that increased expression of HSP10 might be a response designed to protect SOD1(-) cells from oxidative stress, and that the protein may not be involved in protein folding processes in the mitochondria. PPIA catalyzes the cis-trans isomerization of peptidyl-propyl bonds (PPIase) and folds nascent or denatured proteins. Since its expression is induced in response to a wide variety of stressors, including oxidative stress [8], increased expression of PPIA in SOD1(-) cells likely represents a response to the increased oxidative stress associated with the loss of SOD1. In addition to its role in mediating protein folding, it was recently demonstrated that PPIA has antioxidative properties [9] and also contributes to the activation of transcription by stimulating the mitogen-activated protein kinase (MAPK) cascade, which regulates various cellular activities, including gene expression, differentiation, proliferation, and cell survival/apoptosis [10]. PDIA3 is a member of the PDI family of proteins that catalyze disulfide bond formation, reduction, and isomerization of newly synthesized or decomposed proteins in the endoplasmic reticulum (ER). The protein functions as a chaperone and redox catalyst, and its expression is usually upregulated during stress [11]. The upregulation of PDIA3 expression in SOD1(-) cells therefore seems to be an adaptive response to protect cells from the damaging effects of stress. Since CCT is a heterooligomeric molecular chaperone that assists in the folding of actin, tubulin, and other cytosolic proteins [12], upregulation of CCT expression in SOD1(-) cells might assist in the maturation of cytoskeletal proteins needed to counteract the effects of stress. The HSPs and PPIA are known to possess tumor-promoting effects, and overexpression of these proteins has been demonstrated in several types of tumors and pretumoral cells [7-8, 12]. However, the detailed mechanism(s) underlying tumor promotion and the corresponding signaling pathway(s) affected have yet to be determined.

Finally, several proteins involved in cytoskeleton formation were found to be differentially expressed in SOD1(-) cells, including thymosin beta 15, hypothetical protein RCJMB04_5f14 (destrin), and FKBP52. Thymosin beta 15 is an actin-sequestering protein, which prevents actin monomers (G-actin) from binding to actin filaments (F-actin) by binding to the monomers. Increases in the intracellular concentration of thymosin beta have been shown to result in the depolymerization of F-actin [13]. Since destrin is also involved in the depolymerization of F-actin [14], the increased expression of both proteins in SOD1(-) cells probably contributes to destruction of the cytoskeleton. In addition to its role with HSP90 in the intracellular trafficking of GR, it has been reported that FKBP52 specifically and directly interacts with tubulin, resulting in depolymerization of microtubules [15]. Therefore, increased expression of FKBP52 may lead to destruction of the cytoskeleton through the depolymerization of microtubules. On the other hand, since PG, NDK, and CCT are involved in maintenance of the actin cytoskeleton, microtubule polymerization via the production of GTP, and folding of actin and tubulin, respectively, increased expression of these proteins would counteract the destruction of the cytoskeleton brought about by increased expression of thymosin beta 15, destrin, and FKBP52. It has also been reported that thymosin beta is involved in cell survival, and overexpression of the protein has been shown to reduce the levels of phosphorylated c-Jun, which is a member of the MAPK cascade [16]. Since oxidative stress is one of the most potent activators of the MAPK cascade [17], the activation of apoptosis signaling brought about by oxidative stress in SOD1(-) cells might be mitigated by upregulation of thymosin beta 15 expression.

Eventually, the data obtained using the FD-LC-MS/MS proteomic strategy described here may enable us to obtain a more comprehensive understanding of the changes in cellular protein networks that occur in response to various stressors, such as the depletion of SOD1 (Figs. 4 and 5).

## References

1. Zhurinsky J, Shtutman M, Ben-Ze'ev A (2000) Differential mechanisms of LEF/TCF family-dependent transcriptional activation by beta-catenin and plakoglobin. Mol Cell Biol. 20: 4238-4252.
2. Tatro ET, Everall IP, Kaul M, Achim CL (2009) Modulation of glucocorticoid receptor nuclear translocation in neurons by immunophilins FKBP51 and FKBP52: Implications for major depressive disorder. Brain Res. 1286: 1-12.
3. Ren Q, Zhang W, Zhao XF, Wang JX (2008) Gene cloning and expression analysis of ubiquitin derived from Musca domestica. Arch Insect Biochem Physiol. 68: 89-99.
4. Hao Y, Kong X, Ruan Y, Gan H, Chen H et al. (2011) CDK11p46 and RPS8 associate with each other and suppress translation in a synergistic manner. Biochem Biophys Res Commun. 407: 169-174.
5. Kuroda K, Takenoyama M, Baba T, Shigematsu Y, Shiota H et al. (2010) Identification of ribosomal protein L19 as a novel tumor antigen recognized by autologous cytotoxic T lymphocytes in lung adenocarcinoma. Cancer Sci. 101: 46-53.
6. Trotta CR, Paushkin SV, Patel M, Li H, Peltz SW (2006) Cleavage of pre-tRNAs by the splicing endonuclease requires a composite active site. Nature 441: 375-377.
7. Jia H, Halilou AI, Hu L, Cai W, Liu J et al. (2011) Heat shock protein 10 (Hsp10) in immune-related diseases: one coin, two sides. Int J Biochem Mol Biol 2: 47-57.
8. Lee J (2010) Role of Cyclophilin A during Oncogenesis. Arch Pharm Res. 33: 181-187.
9. Hong F, Lee J, Piao YJ, Jae YK, Kim YJ, et al. (2004) Transgenic mice overexpressing cyclophilin A are resistant to cyclosporin A-induced nephrotoxicity via peptidyl-prolyl cis-trans isomerase activity. Biochem Biophys Res Commun. 316: 1073-1080.
10. Satoh K, Nigro P, Berk BC (2010) Oxidative Stress and Vascular Smooth Muscle Cell Growth: A Mechanistic Linkage by Cyclophilin A. Antioxid Redox Signal. 12: 675-682.
11. Turano C, Coppari S, Altieri F, Ferraro A (2002) Proteins of the PDI family: Unpredicted non-ER locations and functions. J Cell Physiol. 193: 154-163.
12. Song XM, Wang XF, Zhuo W, Shi HB, Feng D, et al. (2010) The Regulatory Mechanism of Extracellular Hsp90 alpha on Matrix Metalloproteinase-2 Processing and Tumor Angiogenesis. J Biol Chem. 285: 40039-40049.
13. Chen CY, Li M, Yang H, Chai H, Fisher W, et al. (2005) Roles of thymosins in cancers and other organ systems. World J Surg. 29: 264-270.
14. Moriyama K, Nishida E, Yonezawa N, Sakai H, Matsumoto S, et al. (1990) Destrin, a mammalian actin-depolymerizing protein, is closely related to cofilin. Cloning and expression of porcine brain destrin cDNA. J Biol Chem 265: 5768-5773.
15. Chambraud B, Belabes H, Fontaine-Lenoir V, Fellous A, Baulieu EE (2007) The immunophilin FKBP52 specifically binds to tubulin and prevents microtubule formation. FASEB J. 21: 2787-2797.
16. Choi SY, Kim DK, Eun B, Kim K, Sun W, et al. (2006) Anti-apoptotic function of thymosin-beta in developing chick spinal motoneurons. Biochem Biophys Res Commun. 346: 872-878.
17. Nagai H, Noguchi T, Homma K, Katagiri K, Takeda K, et al. (2009) Ubiquitin-like Sequence in ASK1 Plays Critical Roles in the Recognition and Stabilization by USP9X and Oxidative Stress-Induced Cell Death. Mol Cell. 36: 805-818.
